# Short Multicomponent Group Exercise Intervention Promotes Long-Term Physical Activity Habits among Community-Dwelling Older Adults during COVID-19 Restrictions: A Cohort Study

**DOI:** 10.3390/ijerph192215140

**Published:** 2022-11-17

**Authors:** Marina Arkkukangas, Karin Strömqvist Bååthe, Anna Ekholm, Michail Tonkonogi

**Affiliations:** 1 School of Health and Welfare, Department of Medicine and Sport Sciences, Dalarna University, 791 88 Falun, Sweden; 2 School of Health, Care and Social Welfare, Department of Physiotherapy, Mälardalen University, 721 23 Vasteras, Sweden; 3 Research and Development in Sörmland, Region Sörmland, 632 17 Eskilstuna, Sweden

**Keywords:** training, falls, health and well-being, sedentary behavior

## Abstract

This study investigated whether strength, balance, body mass index, falls self-efficacy, activity levels, self-rated health, and participation in a multicomponent exercise intervention could predict physical activity levels after 5 months of self-quarantine due to the COVID-19 pandemic. This study included baseline data of 200 community-dwelling older adults (79% women, 21% men) with a mean age of 72 years who participated in a randomized controlled trial investigating a multicomponent exercise program, with 7-month follow-up survey data of their physical activity levels. The results showed significant associations with the activity levels at the 7-month follow-up. The activity levels (odds ratio (OR): 2.83, 95% CI: 1.20–6.71), the self-rated health score (2.80, 1.42–5.53), and being allocated to a specific multicomponent group-based exercise program (2.04, 1.04–4.00) showed a significant association with the activity habits at the 7-month follow-up. As this study suggests, besides the physical activity levels and the self-rated health score, participation in a high challenge multicomponent exercise program was significantly associated with physical activity levels at the 7-month follow-up. This study indicates that a relatively short multicomponent group exercise program (6–9 weeks) can motivate individuals to sustain their own training and activity levels even several months after the program has been paused or terminated. Identifying older adults’ physical activity levels and self-rated health scores and prescribing multicomponent group-based exercise programs to promote sustained physical activity habits may be a successful alternative to provide for older adults in the future.

## 1. Introduction

Globally, the knowledge on the coronavirus disease (COVID-19) and its impact on people’s health is required. This is especially important among older adults, as they are one of the main at-risk groups for COVID-19 [1]. During the COVID-19 pandemic, the importance of physical activity has been highlighted, and older adults’ participation in physical activity and exercise is reported to be generally low [2]. Recently, a systematic review confirmed that the level of physical activity among older adults during the COVID-19 pandemic was significantly reduced [3]. A reduction in their physical activity levels may lead to decline in physical fitness and an increased sedentary lifestyle, which are common risk factors commonly resulting in a decline in physical function [4,5]. Further, a decline in physical function is also related to increased frailty in this population [6]. In addition, isolation during a pandemic affects one’s social life negatively, leading to a higher risk of loneliness and depression [7]. Therefore, isolation and inactivity due to a pandemic will certainly have a great impact on older adults’ activity levels and health. In mid-March 2020, all of the adults in Sweden over 70 years of age were strongly encouraged to self-quarantine. All group activities such as sports, church groups, and retirement associations’ social and cultural activities were paused for this age group. In Sweden, people in general were used to following government guidelines so although it was not a strict lockdown with legal punishments, the recommendations were generally followed by the population.

Physical activity and exercise as means of achieving a healthy lifestyle among older adults have been rigorously documented [8]. Up to the present date, there is a modest but growing volume of evidence of the effective interventions promoting physical activity among older adults. However, there is still a need for the long-term follow ups of such interventions. In addition, older adults’ propensity to perform behavioral changes and their adherence to the current recommendations of physical activity and exercise is generally low, and these decline over time, leading to an inevitable decline in physical functions [8]. Research studies have shown that balance and strength losses may occur within 4–20 weeks of detraining, independent of the prior resistance training intensity. Efforts to promote the adherence to it have been investigated, and the researchers have highlighted the importance of including behavioral, physical, and social factors in the complex interplay of the interaction between the physical activity and health-related behavioral changes [9,10]. Thus, even though the knowledge on the benefits of physical activity and exercise has been reported, the adherence to such interventions over time still remains a challenge [11,12]. Furthermore, periods of isolation and inactivity may lead to a decline in physical function, such as decreased muscle strength and balance in older adults, which are highly associated with a higher risk of falls and fall-related injuries; exercises targeting these functions are suggested to be effective in counteracting this decline [13]. Studies investigating the effects of sedentary and social isolation among older adults due to the COVID-19 pandemic have confirmed the associations with decreased physical functioning and the fall outcomes [14].

To address falls and fall-related injuries, commonly used fall-prevention exercise programs have focused on strengthening the muscles, improving the balance, and to some extent, getting down to and up from the floor [15,16]. However, the importance of including highly challenging strength and balance exercises and falling techniques, which are integrated with behavioral, physical, and social factors, in such programs are important [16]. Since physical activity is known to be associated with several health benefits [17], it became important during the COVID-19 pandemic to explore these benefits among this self-isolated group of older adults. Therefore, this cohort study aimed to investigate whether strength, balance, body mass index (BMI), falls self-efficacy, activity levels, self-rated health, and participation in a highly challenging multicomponent fall-prevention exercise intervention program could predict the physical activity levels after a 7-month follow-up following the COVID-19-related isolation in Sweden.

## 2. Materials and Methods

This was a prospective cohort study based on the baseline data from a randomized controlled trial (RCT) including one treatment and one control group. In the RCT, to address the falls and its highly unprioritized threat to older adult’s health, a fall prevention research project was started in January 2020 in Sweden. The researchers planned to investigate the practice of fall prevention exercises and the rate of learning safe falling techniques among adults over 65 years of age. Following the findings of a feasibility study on the Judo4Balance program in 2018 [18], the intervention was designed as a 12-week program with progressive exercise challenges. The control group received no intervention during the study. However, after the data collection was completed and the study was finalized, the participants of the control group were offered to participate in the Judo4Balance program.

The RCT project included 200 older adults, who were randomized to either an intervention or control group. The RCT aimed to investigate the short-term effects of a 12-week judo-inspired multicomponent group-based exercise program (Judo4Balance) regarding physical functions, falls, fall-related self-efficacy, and learning falling techniques [19]. The intervention was a standardized multicomponent Judo4Balance exercise program, which was a weekly 50–60 min group exercise program that was led by two licensed Judo4Balance instructors. The program consisted of three main blocks:

(1) Practicing fall techniques and strength exercises, body awareness, mobility training, building up the load of the resistance in the muscles, tendons, joints, and skeleton, as well as highly challenging exercises to train the balance of the participants by performing movements that are not usually carried out in everyday life activities, e.g., getting up and down from the floor.
(2) Continuing the fall techniques and strength exercises, increasing the load in the strength exercises, challenging the balance and coordination ability, employing a greater range of movements in the exercises, and possibly, in the power in the exercises, and continuing to build up the load resistance in the muscles, tendons, joints, and skeleton.
(3) Training the ability to develop power (fast power), power in the strength exercises, and possibly, in training in the fall techniques, challenging one’s balance with increased difficulty.

The Judo4Balance is a structured and standardized program, and all of the judo instructors were well acquainted with the program as well as experienced in performing the group exercises. Each training session has a similar pattern, which started with a warm-up, proprioception and breakfall techniques, strength training, and then, a cool down and relaxation period. Many of the exercises were performed in pairs (a common practice in judo).

The RCT intervention started in mid-January 2020, and it was abruptly interrupted and put on hold in March 2020. Since the intervention had been running for half of the time (6–9 weeks), there was an interest to further investigate and follow up the physical activity habits in a merged study group following a 7-month follow-up, after the baseline measures.

### 2.1. Data Source

Due to the COVID-19 outbreak in March 2020, deviations from the original RCT study protocol were made, resulting in the present study. The participants were recruited through social media and contact was made with retirement organizations. All of the first 200 participants who showed interest in the study accepted their participation in nine different regions of the country in large, medium, and small cities in Sweden.

The inclusion criteria were as follows: being over 65 years of age, having the ability to understand written and verbal Swedish language, and being able to walk independently. The exclusion criteria included having uncontrollable high blood pressure or a retinal detachment, which did not allow them to complete the exercise.

The first 200 participants who showed interest in the study were contacted by phone and given information about the study by four research assistants in the research group. If they were willing to participate in the study, they were contacted by three test leaders to obtain the baseline measurements. If the participants met the study inclusion criteria, their informed consent was obtained, and they were then randomized into one of the two groups.

Since the baseline physical and psychological measurements in the RCT were conducted in January 2020, this study reported the baseline data and additional data from a follow-up survey on their physical activity habits which were collected in August 2020 because older adults were restricted from leaving their homes during this period (during the first wave of the pandemic). The follow-up survey included questions that were mainly on the physical activity levels during the 6 months prior to the study.

### 2.2. Measurements

All of the independent variables included in this study were measured at the baseline in the main RCT, and they were predictive of the 7-month follow-up physical activity. The test leaders who performed the baseline tests were blinded to which group the participants were allocated. The test leaders were familiar with the tests that were included in the study, and they were all licensed Judo4Balance instructors. The dependent variable was the physical activity habits.

#### 2.2.1. Dependent Variable

In the survey, the Frändin/Grimby activity scale was used [20]; the scale is commonly used to assess older adults’ physical activity levels. The scale includes the following six levels of physical activity: (1) hardly any physical activity, (2) mostly sitting, sometimes a walk, and light household activities, (3) light physical exercise for around 2–4 h a week, (4) moderate exercise for 1–2 h a week, (5) moderate exercise for at least 3 h a week, and (6) strenuous or very strenuous regular exercise several times a week, where the physical exertion is high. The participants self-rated their physical activity levels during the past 6 months. The scale has been shown to be valid for assessing the physical activity levels among older adults [20]. The Frändin/Grimby activity scale scores were also measured at the baseline.

#### 2.2.2. Independent Variables

The first model included nine independent variables: age, sex, the treatment group, the activity level, the self-rated health score using the Euro Quality of life visual analog (EQ VAS) scale, BMI, the physical performance using the SPPB scale, the Mini-BESTest, and the Falls Self-Efficacy Scale Swedish version (FES-S).

The EQ VAS was used to measure the self-rated health score on a vertical VAS. The scale is labeled from “The best health you can imagine” to “The worst health you can imagine,” with a score ranging from 0 to 100. The VAS is a quantitative measure of the health outcomes, and it reflects the patient’s own judgment [21]. The BMI was measured at the baseline since an association between the BMI and the physical functioning in older adults has been reported [22]. The physical performance was measured using the SPPB scale, which evaluates the functioning of the lower extremities in older persons, which are the standing balance, the gait speed, and repetitive chair standing actions [23]. The tasks were graded on a four-point scale, with a maximum score of 12 points; the maximum score indicates the best physical performance. The balance was measured using the Mini-BESTest, which includes 14 different tasks along the following four subscales: anticipatory, reactive postural control, sensory orientation, and dynamic gait [24]. All of the tasks were graded from 0 to 2 points, with a total maximum score of 28 points for each task. To measure the confidence in a person’s ability to perform various daily activities without falling, the FES-S was used. The scale includes 13 items, with a total score of 130. The scale score ranges from 0 to 10, with 0 representing a low fall self-efficacy and 10 representing a high fall self-efficacy [25].

### 2.3. Statistical Analysis

The baseline characteristics for the exercise and control groups are presented as the mean (age), proportion (sex and BMI), and median (FES-S, Mini-BESTest, and SPPB). The difference between the groups was analyzed using a t test, a chi-square test, and a Mann–Whitney U test. A logistic regression was used to estimate the associations between the physical activity and several physical and behavioral factors which were collected at the baseline, after adjusting for sex and age. In the logistic regression analysis, the dependent variable, the physical activity level, was categorized into low-to-light exercise (1–3) and moderate-to-hard exercise groups (4–6). The independent variables were grouped as follows: physical activity, low-to-light exercise (1–3), and moderate-to-strenuous exercise (4–6), which were calculated from the median values in this study; self-rated health (using the EQ VAS), was categorized into between ≤80 and >80 [18]; BMI, was categorized into from not overweight (<25) to overweight (≥25); SPPB, was categorized into between <10 and ≥10 [26]; Mini-BESTest, was categorized into between <22 and ≥22 [27]; the FES-S scores were grouped for the women and the men into low (scores less than 124 vs. 126, respectively) and high groups (score equal to or higher than 118 vs. 105, respectively) [28]. The treatment group was included as an independent variable to investigate whether the half-time participation in the high challenge exercise program (Judo4Balance) was associated with physical activity habits. The *p*-values were set at a significance level of 0.05. The results are reported as odds ratios (ORs) and at 95% confidence intervals (95% CIs). All of the analyses were performed using IBM SPSS Statistics for Windows, version 22.0 (IBM Corp., Armonk, NY, USA).

### 2.4. Ethics

The study was conducted in accordance with the principles of the Declaration of Helsinki. The RCT including the additional components that were performed under the present study were approved by Swedish Ethical Review Authority Dnr: 2019-03048. Each participant voluntarily provided written informed consent before participating in both the RCT and the present study. The participants received no compensation for participating in the study. The study and study protocol are registered at clinical trials gov. Clinical trial registration: NCT04061785.

## 3. Results

In this 7-month follow up cohort study, 199 participants (one dropout from the control group) received the follow-up questionnaire; 175 men and women aged 65 years and above responded to the survey. The response rate was 88%. The descriptions of the participants and their baseline values are presented in Table 1. The mean age was 72 years. There were no significant differences between the groups in any of the baseline measurements (Table 1).

The results of the regression models are presented in Table 2 and Table 3. All of the independent variables were included in the first regression model. Three variables displayed significant values at a 0.05 significant level: the intervention group, the activity levels, and the self-rated health score (see Table 3). Table 3 shows that factors that were independently associated with physical activity levels were the physical activity (OR: 2.83, 95% CI: 1.20–6.71) and the self-rated health score (OR: 2.80, 95% CI: 1.42–5.53). In addition, being allocated to a specific exercise treatment group (OR: 2.04, 95% CI: 1.04–4.00) was also significantly predictive of the physical activity levels.

## 4. Discussion

During the COVID-19 pandemic, it has been suggested that physical and mental health are important aspects to address because of their protective effects against serious health conditions among older adults [29]. The results from this study showed that physical activity levels and self-rated health were significantly associated with activity levels during the 7-month follow-up period in older adults who were isolated due to the COVID-19 pandemic in Sweden. Although the benefits of physical activity and exercise are well known, adherence to such activities remains a challenge [30,31]. Therefore, the behavioral, physical, and social factors that are reported in our study are considered to be important for sustainable activity habits. These are especially important because physical inactivity over a period of weeks and months can lead to immune system dysfunction which can worsen the pathophysiology of conditions, including cardiovascular disease, cancer, and inflammatory disorders, which are common in older adults [32,33]. In addition, engaging in a highly challenging multicomponent group-based fall-prevention exercise program, which includes behavioral, physical, and social factors, such as the intervention Judo4Balance program displayed a significant association with high activity levels at the follow-up. These results were considered to be valuable because the short exercise duration (half-time intervention) in this study, with there being a minimum of six sessions over a period of six weeks, was significantly associated with the adherence to physical activity levels at the follow-up in this study. Despite the current knowledge of the impact of physical activity on older adults’ health [2,34], there still is a lack of effective long-term exercise regimens [35]. The research has suggested that performing exercise for at least 10 weeks may positively affect the balance function [36]. This suggests that the Judo4balance program that was used in the RCT may be an effective program in the promotion of physical activity for older adults.

Furthermore, participation in social activities and group-based exercise is considered to be important for the health of older adults [37,38], and it may lead to increased exercise self-efficacy [37]. In addition, group-based fall-preventive exercise is favored as opposed to individually performed exercise [38], and highly challenging exercise programs are recommended and warranted. This indicates the importance of providing and promoting the combination of behavioral/physical and social exercise forms for older adults, especially when one is considering the prevention of physical decline, including the prevention of falls, as well as for the sustainability of the exercise. Thus, these are highlighted, particularly to address the occurrence of involuntarily isolation due to different circumstances in life, in this case, the COVID-19 pandemic. To understand human behaviors from a theoretical context, Bandura’s social cognitive theory (SCT) can be applied [39]. The interaction between the behavioral and personal and environmental factors, which influence the direction and actions that are taken, are central to promoting health-related behaviors. Therefore, these domains should be highlighted; based on our results, all of the aspects of the behavioral, physical/personal, and especially social factors are important to be considered when one is striving for long-term health-related behaviors [39].

Surprisingly, the physical measures in this study, the SPPB and Mini-BESTest, revealed no association with the physical activity levels at the follow-up. However, this older adult group was, in general, a highly functional one, with a median score of 11/12 on the SPPB scale and score of 22/28 on the Mini-BESTest. The normative values of these tests, in the same age group, are suggested as follows: for the Mini-BESTest, a score of ≤21 differentiates those with and without postural response deficits with a sensitivity of 89% and specificity of 81% [27]. For the SPPB, a score of 10–12 has been suggested as a normative score for highly functional older adults [26]. Thus, these findings are important in providing knowledge about examining and addressing the behavioral and social factors as well as the physical status, when one is aiming to achieve sustainable exercise habits. This has been addressed previously in a study investigating the adherence to fall-preventive exercise, where behavioral factors and motivational support significantly predicted the adherence to exercise [40]. The group-based Judo4Balance program targets and includes the common risk factors for falls, such as balance, strength, endurance, and flexibility, and this program also covers other physical and mental benefits that are derived by engaging in physical activity and exercise [41]. As there is a known issue with non-participation in physical activity and exercise especially at higher exercise levels where the health benefits among older adults are ensured, and the social, physical, and behavioral factors need to be addressed [12,40,41]. Therefore, it is of utmost importance to consider these three factors that we included in this study in interventions, when one is striving for sustainable physical activity habits among older adults.

The narrow range of the independent variables that were included in the regression models in this study could be considered as a limitation of it. Nonetheless, the variables that were included in the final model were judged to be clinically important, and the number of variables was appropriate considering the relatively small study sample size. We are also aware that the sample is not representative of a broader older population, since all of the participants agreed to participate in the highly challenging exercise intervention. However, since the preventive action needed to have occurred early in life to ensure a protective support, we believe that this study contributes to the knowledge about what necessary action needs to be addressed in order to achieve this prevention. This study investigated both the exercise and control groups; since there was no significant differences which we reported between these groups at the baseline, we considered that a merged group could provide more rigorous data for our analysis. Despite the limitations, we believe that the knowledge that we provided based on the findings of this study suggests the need to further explore the behavioral factors, physical factors, and participation in group-based exercise in community-dwelling older adults. In addition, continuing exercising with Judo4Balance during the 7-month break was considered to be highly unlikely since the program Judo4Balance was only offered in “live” session or on zoom in real time with an instructor. Videos on other “regular” judo practices would be very hard for the participants to follow or to get any systematic training from.

## 5. Conclusions

The results of this study indicate that physical activity levels, self-rated health scores, and the participation in a shorter multicomponent group-based exercise intervention seem to have a motivating effect for the participants to stay physically active even when the group program had to come to a pause. This knowledge may be important when one is supporting older adults in sustaining a healthy and an active lifestyle, achieving behavioral changes and adherence to physical activity. Sustaining a healthy and active lifestyle has proven to be difficult during the COVID-19 pandemic. Our results can contribute to the development of preventive strategies for the older population.

## Figures and Tables

**Table 1:** Baseline characteristics.

Participant Demographics	Total	Exercise *n* = 100	Control *n* = 99	*p*-Value
Age (mean, SD)	72 (4.9)	71.5 (4.5)	72.7 (5.2)	0.083
Gender (female/male)	79%/21%	76%/24%	82%/18%	0.302
BMI ≥ 25 kg/m^2^	52%	54%	50%	0.668
Pysical activity level (1–6), median (min–max)	4 (1–6)	4 (2–6)	4 (1–6)	0.519
SPPB (0–12), median (min–max)	11 (2–12)	11 (2–12)	11 (5–12)	0.098
Mini-BESTest (0–28), median (min–max)	22 (4–27)	22 (11–27)	21 (4–27)	0.191
FES-S (0–130), median (min–max)	127 (52–130)	126.5 (52–130)	127 (65–130)	0.391
Self-rated health, (0–100), median (min–max)	80 (25–100)	80 (25–100)	80 (36–100)	0.898

Abbreviations: SD, standard deviation; BMI, body mass index; SPPB, short physical performance battery; FES-S, Falls Efficacy Scale Swedish version.

**Table 2:** First logistic regression model showing independent variables independently associated with the level of physical activity, *n* = 175.

Predictors	OR	95% CI for OR
Age	0.99	0.90–1.05
Gender	0.87	0.36–2.09
Intervention group	2.30 *	1.14–4.63
Physical activity (at baseline)	3.03 *	1.23–7.44
Self-rated health, EQ VAS scale	2.84 *	1.37–5.90
BMI	1.75	0.86–3.58
SPPB	0.63	0.26–1.52
Mini-BESTest	1.06	0.48–2.41
FES-S	0.97	0.44–2.15

Abbreviations: CI, confidence interval; OR, odds ratio; SPPB, short physical performance battery; FES-S, Falls Efficacy Scale Swedish version. *, significant value ≤ 0.05.

**Table 3:** Final logistic regression model showing independent variables associated with the level of physical activity, *n* = 175.

Predictors	OR	95% CI for OR
Age	0.98	0.91–1.05
Gender	0.97	0.42–2.24
Intervention group	2.04 *	1.04–4.00
Physical activity (at baseline)	2.83 *	1.20–6.71
Self-rated health, EQ VAS scale	2.80 *	1.42–5.53

Abbreviations: CI, confidence interval; OR, odds ratio; *, significant value ≤ 0.05.

## Data Availability

The data presented in this study are available upon request from the corresponding author. The data are not publicly available because of privacy or ethical restrictions.

## References

[1] Sharma A. (2021). Estimating older adult mortality from COVID-19. J. Gerontol. B Psychol. Sci. Soc. Sci..

[2] World Health Organization (2020). Who Guidelines on Physical Activity and Sedentary Behaviour. https://apps.who.int/iris/bitstream/handle/10665/336656/9789240015128-eng.pdf?sequence=1&isAllowed=y.

[3] Oliveira M.R., Sudati I.P., Konzen V.M., de Campos A.C., Wibelinger L.M., Correa C., Miguel F.M., Silva R.N., Borghi-Silva A. (2022). COVID-19 and the impact on the physical activity level of elderly people: A systematic review. Exp. Gerontol..

[4] Baldwin C.E., Phillips A.C., Edney S.M., Lewis L.K. (2020). Recommendations for older adults’ physical activity and sedentary behaviour during hospitalisation for an acute medical illness: An international Delphi study. Int. J. Behav. Nutr. Phys. Act..

[5] Kehler D.S. (2018). The impact of sedentary and physical activity behaviour on frailty in middle-aged and older adults. Appl. Physiol. Nutr. Metab..

[6] Taylor J., Walsh S., Kwok W., Pinheiro M.B., de Oliveira J.S., Hassett L., Bauman A., Bull F., Tiedemann A., Sherrington C. (2021). A scoping review of physical activity interventions for older adults. Int. J. Behav. Nutr. Phys. Act..

[7] Krendl A.C., Perry B.L. (2021). The Impact of Sheltering in Place during the COVID-19 Pandemic on Older Adults’ Social and Mental Well-Being. J. Gerontol. B Psychol. Sci. Soc. Sci..

[8] Sherrington C., Fairhall N., Kwok W., Wallbank G., Tiedemann A., Michaleff Z.A., Ng C.A.C.M., Bauman A. (2020). Evidence on physical activity and falls prevention for people aged 65+ years: Systematic review to inform the WHO guidelines on physical activity and sedentary behaviour. Int. J. Behav. Nutr. Phys. Act..

[9] Lachman M.E., Lipsitz L., Lubben J., Castaneda-Sceppa C., Jette A.M. (2018). When Adults Don’t Exercise: Behavioral Strategies to Increase Physical Activity in Sedentary Middle-Aged and Older Adults. Innov. Aging.

[10] John J.M., Haug V., Thiel A. (2020). Physical Activity Behavior from a Transdisciplinary Biopsychosocial Perspective: A Scoping Review. Sports Med. Open..

[11] Spiteri K., Broom D., Hassan Bekhet A., Xerri de Caro J., Laventure B., Grafton K. (2019). Barriers and Motivators of Physical Activity Participation in Middle-Aged and Older Adults—A Systematic Review. J. Aging Phys. Act..

[12] Martin K.A., Bowen D.J., Dunbar-Jacob J., Perri M.G. (2000). Who Will Adhere? Key Issues in the Study and Prediction of Adherence in Randomized Controlled Trials. Control. Clin. Trials.

[13] Sherrington C., Fairhall N.J., Wallbank G.K., Tiedemann A., Michaleff Z.A., Howard K., Clemson L., Hopewell S., Lamb S.E. (2019). Exercise for Preventing Falls in Older People Living in the Community. Cochrane Database of Systematic Reviews. http://doi.wiley.com/10.1002/14651858.CD012424.pub2.

[14] Hoffman G.J., Malani P.N., Solway E., Kirch M., Singer D.C., Kullgren J.T. (2022). Changes in activity levels, physical functioning, and fall risk during the COVID-19 pandemic. J. Am. Geriatr. Soc..

[15] Gardner M.M. (2001). Practical implementation of an exercise-based falls prevention programme. Age Ageing.

[16] Skelton D., Dinan S., Campbell M., Rutherford O. (2005). Tailored group exercise (Falls Management Exercise—FaME) reduces falls in community-dwelling older frequent fallers (an RCT). Age Ageing.

[17] Galloza J., Castillo B., Micheo W. (2017). Benefits of Exercise in the Older Population. Phys. Med. Rehabil. Clin. N. Am..

[18] Arkkukangas M., Strömqvist Bååthe K., Hamilton J., Ekholm A., Tonkonogi M. (2020). Feasibility of a novel Judo4Balance—Fall preventive exercise programme targeting community-dwelling older adults. J. Frailty Sarcopenia Falls.

[19] Arkkukangas M., Strömqvist Bååthe K., Ekholm A., Tonkonogi M. (2022). High Challenge Exercise and Learning Safe Landing Strategies among Community-Dwelling Older Adults: A Randomized Controlled Trial. Int. J. Environ. Res. Public Health.

[20] Frändin K., Grimby G. (2007). Assessment of physical activity, fitness and performance in 76-year-olds. Scand. J. Med. Sci. Sport..

[21] Szende A., Janssen B., Cabases J. (2014). Self-Reported Population Health: An International Perspective Based on EQ-5D.

[22] Woo J., Leung J., Kwok T. (2007). BMI, Body Composition, and Physical Functioning in Older Adults. Obesity.

[23] Guralnik J.M., Simonsick E.M., Ferrucci L., Glynn R.J., Berkman L.F., Blazer D.G., Scherr P.A., Wallace R.B. (1994). A Short Physical Performance Battery Assessing Lower Extremity Function: Association with Self-Reported Disability and Prediction of Mortality and Nursing Home Admission. J. Gerontol..

[24] Franchignoni F., Horak F., Godi M., Nardone A., Giordano A. (2010). Using psychometric techniques to improve the Balance Evaluation Systems Test: The mini-BESTest. J. Rehabil. Med..

[25] Fugl-Meyer A.R., Hellström K., Lindmark B., Wahlberg B. (2003). Self-efficacy in relation to impairments and activities of daily living disability in elderly patients with stroke: A prospective investigation. J. Rehabil. Med..

[26] Guralnik J.M., Ferrucci L., Pieper C.F., Leveille S.G., Markides K.S., Ostir G.V., Studenski S., Berkman L.F., Wallace R.B. (2000). Lower Extremity Function and Subsequent Disability: Consistency Across Studies, Predictive Models, and Value of Gait Speed Alone Compared with the Short Physical Performance Battery. J. Gerontol. Biol. Sci. Med. Sci..

[27] King L.A., Priest K.C., Salarian A., Pierce D., Horak F.B. (2012). Comparing the Mini-BESTest with the Berg Balance Scale to Evaluate Balance Disorders in Parkinson’s Disease. Park. Dis..

[28] Andersson S., Larsson S. (2010). Förslag till rekommenderade referensvärden för Falls Efficacy Scale (Swedish version) för friska äldre. http://uu.diva-portal.org/smash/record.jsf?pid=diva2%3A321779&dswid=7693.

[29] Cunningham C., O’Sullivan R., Caserotti P., Tully M.A. (2020). Consequences of physical inactivity in older adults: A systematic review of reviews and meta-analyses. Scand. J. Med. Sci. Sports.

[30] Collado-Mateo D., Lavín-Pérez A.M., Peñacoba C., Del Coso J., Leyton-Román M., Luque-Casado A., Gasque P., Fernández-del-Olmo M.Á., Amado-Alonso D. (2021). Key Factors Associated with Adherence to Physical Exercise in Patients with Chronic Diseases and Older Adults: An Umbrella Review. Int. J. Environ. Res. Public Health.

[31] Papp M.E., Grahn-Kronhed A., Rauch Lundin H., Salminen H. (2022). Changes in physical activity levels and relationship to balance performance, gait speed, and self-rated health in older Swedish women: A longitudinal study. Aging Clin. Exp. Res..

[32] Damiot A., Pinto A.J., Turner J.E., Gualano B. (2020). Immunological Implications of Physical Inactivity among Older Adults during the COVID-19 Pandemic. Gerontology.

[33] Duggal N.A., Niemiro G., Harridge S.D.R., Simpson R.J., Lord J.M. (2019). Can physical activity ameliorate immunosenescence and thereby reduce age-related multi-morbidity?. Nat. Rev. Immunol..

[34] Dohrn I.M., Hagströmer M., Hellénius M.L., Ståhle A. (2017). Short- and Long-Term Effects of Balance Training on Physical Activity in Older Adults with Osteoporosis: A Randomized Controlled Trial. J. Geriatr. Phys. Ther..

[35] Ehrari H., Larsen R.T., Langberg H., Andersen H.B. (2020). Effects of Playful Exercise of Older Adults on Balance and Physical Activity: A Randomized Controlled Trial. J. Popul. Ageing.

[36] Komatsu H., Yagasaki K., Saito Y., Oguma Y. (2017). Regular group exercise contributes to balanced health in older adults in Japan: A qualitative study. BMC Geriatr..

[37] Huang W.-Y., Huang H., Wu C.-E. (2022). Physical Activity and Social Support to Promote a Health-Promoting Lifestyle in Older Adults: An Intervention Study. Int. J. Environ. Res. Public Health.

[38] Gawler S., Skelton D.A., Dinan-Young S., Masud T., Morris R.W., Griffin M., Kendricke D., Iliffe S. (2016). Reducing falls among older people in general practice: The ProAct65+ exercise intervention trial. Arch. Gerontol. Geriatr..

[39] Bandura A. (1986). Social Foundations of thought and Action: A Social Cognitive Theory.

[40] Arkkukangas M., Söderlund A., Eriksson S., Johansson A.C. (2018). One-Year Adherence to the Otago Exercise Program with or without Motivational Interviewing in Community-Dwelling Older Adults. J. Aging Phys. Act..

[41] Taylor D. (2014). Physical activity is medicine for older adults. Postgrad. Med. J..

